# Caregivers with Cancer Patients: Focus on Hispanics

**DOI:** 10.3390/cancers15030626

**Published:** 2023-01-19

**Authors:** Jasbir Bisht, Priyanka Rawat, Ujala Sehar, P. Hemachandra Reddy

**Affiliations:** 1 Department of Pediatrics, Texas Tech University Health Sciences Center, Lubbock, TX 79430, USA; 2 Department of Internal Medicine, Texas Tech University Health Sciences Center, Lubbock, TX 79430, USA; 3 Department of Speech, Language and Hearing Sciences, School Health Professions, Texas Tech University Health Sciences Center, Lubbock, TX 79430, USA; 4 Department of Public Health, School of Population and Public Health, Texas Tech University Health Sciences Center, Lubbock, TX 79430, USA; 5 Neurology, Departments of School of Medicine, Texas Tech University Health Sciences Center, Lubbock, TX 79430, USA; 6 Nutritional Sciences Department, College of Human Sciences, Texas Tech University, Lubbock, TX 79409, USA

**Keywords:** cancer, aging population, caregivers, metastatic, resisting cell death, cell proliferation

## Abstract

**Simple Summary:**

Cancer is a disease in which cells divide abnormally and damage body tissues. Cancer is one of the leading causes of death, and due to the interplay of socioenvironmental, behavioral, and biological factors, there are well-established differences in cancer incidence and outcomes by race and ethnicity in the US. Hispanics have a lower incidence of cancer as compared to whites, but the overall trend of fewer cancer screenings in this ethnic group can result in diagnostic and treatment delays and higher death rates. Hispanic cultural values influence their health behaviors and the choice of care for their loved ones. In this review, we focus on Hispanics to discuss cancer risk factors among them, the caregiving aspects, and the identification of interventions to reduce Hispanic caregivers’ burden.

**Abstract:**

Cancer is a public health concern and causes more than 8 million deaths annually. Cancer triggers include population growth, aging, and variations in the prevalence and distribution of the critical risk factors for cancer. Multiple hallmarks are involved in cancer, including cell proliferation, evading growth suppressors, activating invasion and metastasis, resisting cell death, enabling replicative immortality, reprogramming energy metabolism, and evading immune destruction. Both cancer and dementia are age-related and potentially lethal, impacting survival. With increasing aging populations, cancer and dementia cause a burden on patients, family members, the health care system, and informal/formal caregivers. In the current article, we highlight cancer prevalence with a focus on different ethnic groups, ages, and genders. Our article covers risk factors and genetic causes associated with cancer and types of cancers and comorbidities. We extensively cover the impact of cancer in Hispanics in comparison to that in other ethnic groups. We also discuss the status of caregivers with cancer patients and urgent needs from the state and federal support for caregivers.

## 1. Introduction

**Figure 1:**
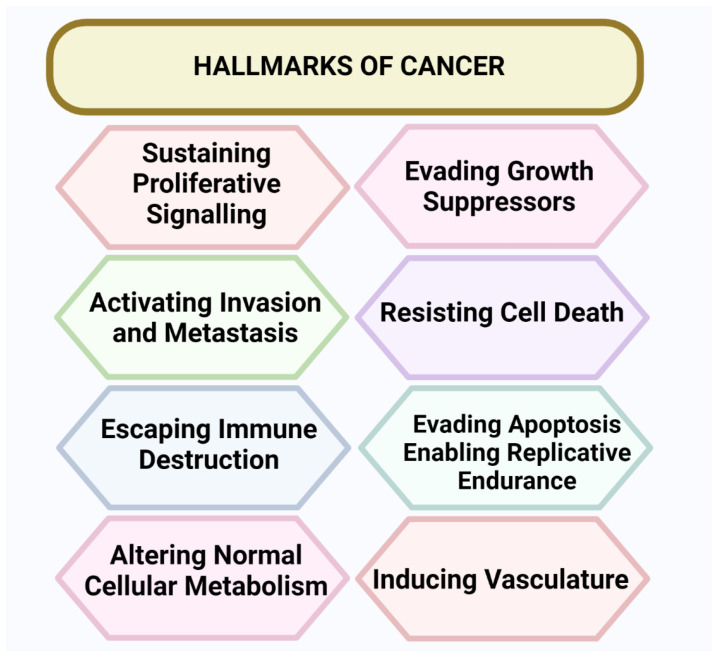
Hallmarks of cancer. There are eight hallmarks of cancer, including developing capabilities to sustain cell proliferative signaling, evading growth suppressors, activating invasion and metastasis, inducing angiogenesis, resisting cell death, enabling replicative immortality, reprogramming energy metabolism, and evading immune destruction.

Cancer cases are rapidly increasing worldwide [1]. Cancer is the second leading cause of death in the United States (USA) [2]. Cancer is a worldwide health concern and causes more than 8 million deaths annually [3]. The cancer triggers, including population growth, aging, and variations in the prevalence and distribution of the critical risk factors for cancer, are associated with socioeconomic conditions [4]. Several lines of evidence have shown that tumorigenesis in humans is a combined multistep process, and that these steps indicate genetic alterations that drive the progressive alterations in normal human cells into highly malignant byproducts [5]. Cancer is a disease caused by the rampant division of cells and disorderly growth. Cancer can begin in any part of the body. Normal cells grow and increase through a cell division mechanism, while cancerous cells grow without a signal, invade the tissues, and disseminate to other parts of the body [6]. There are eight hallmarks of cancer: developing capabilities to sustain cell proliferative signaling, evading growth suppressors, activating invasion and metastasis, inducing angiogenesis, resisting cell death, enabling replicative immortality, reprogramming energy metabolism, and evading immune destruction [7], as shown in Figure 1.

In 2022, Hispanic or Latino residents will likely surpass non-Hispanic whites as the predominant population [8]. Cancer surveillance data are generally only accessible to the Hispanic population, masking significant heterogeneity among Hispanic groups [9]. The Hispanic population, compared to other major racial and ethnic groups, has the highest prevalence of important, possibly modifiable cancer risk factors, including type 2 diabetes mellitus (T2DM) and obesity [10]. Hispanics are diagnosed with advanced stages of disease and have a lower quality of life after receiving a cancer diagnosis than non-Hispanic whites. Multiple factors, including social, cultural, behavioral, and biological, affect the prognosis of cancer. However, in the oncology field, researchers have paid attention to behavioral and psychosocial aspects of non-Hispanic whites, but for Hispanics, our understanding of the complex array of prognostic variables in Hispanics is limited [11]. The Hispanic community also suffers from significant health disparities compared to non-Hispanic whites (NHWs) [12]. Cancer affects individuals with cancer and their caregivers, partners, friends, and family members [13,14]. Cancer treatment takes place in an outpatient setting and requires that most of the care to individuals with cancer is provided by family caregivers [15]. This shift from a hospital facility to an outpatient setting increases caregivers’ caregiving burden. In the last few years, because of the aggressive treatment of cancer [16], individuals have felt the need for caregivers to manage pain, nausea, and other detrimental effects of cancer. With the increasing incidence and survival rates of cancer, more people are living with cancer, which increases the responsibilities of informal caregivers and leads to a higher caregiver burden, depression, and low quality of life [17]. 

Our article aims to highlight the prevalence of cancer both globally and in the US. Our article also covers the challenges faced by Hispanic caregivers of cancer patients, interventions for caregivers, and techniques for validating the interventions. Our critical analysis of published findings can be used to help academics and healthcare professionals to create therapies and supportive techniques for Hispanic caregivers of cancer patients. 

## 2. Current Status of Cancer Worldwide

According to the Global Cancer Observatory, globally,18,094,716 cancer cases were diagnosed in 2020. The age-standardized rate for all cancers (apart from non-melanoma skin cancer) for men and women was 190 per 100,000 in 2020. A summary of global cancer data from the top five countries is presented in Table 1. The rate was more significant for men (206.9 per 100,000) than for women (178.1 per 100,000). Some of the interesting findings on the prevalence of cancer worldwide are presented below:

- The most significant cancer rate for both men and women combined was in Denmark at 334.9 people per 100,000.
- The highest cancer rate in men was found in Hungary at 371 per 100,000.
- The leading cancer rate in women was in Denmark at 328.3 per 100,000.
- The highest rate of cancer deaths for both men and women combined was in Mongolia at 175.9 individuals per 100,000.
- The highest death rate from cancer in men was in Mongolia at 224.3 per 100,000.
- The highest death rate from cancer in women was in Zimbabwe at 142.9 women per 100,000.

Corresponding to the International Agency for Research on Cancer, by 2040, the worldwide cancer burden is expected to expand to 27.5 million new cases of cancer and 16.3 million cancer deaths solely due to the growth and aging of the population. 

## 3. Current Cancer Status in the United States

According to the American Cancer Society (ACS), the risk of individuals dying from cancer in the United States has been reduced over the past 28 years. From 1991 to 2019, the death rate peaked and has now fallen by 32% combined for men and women. According to Centers for Disease Control and Prevention (CDC) data, rural America has shown a slower decline in cancer death rates (1% per year) as compared to urban America (1.6% per year). According to Cancer Facts & Figures 2022, in 2022 the estimated number of new patients diagnosed with cancer was 1.9 million, and 609,360 cancer deaths are projected in the US. In Texas, the projected new cases in 2022 were 139,320, and the estimated deaths were 43,490. According to 2020 census evaluations from the US Census Bureau, 62 million Americans, or 19% of the continental US and Hawaii population, identified themselves as Hispanic and Latino. Moreover, more than 3 million Hispanic Americans reside in US territory. In the continental United States and Hawaii in 2021, around 46,500 cancer deaths and 176,600 new cancer cases were projected among the Hispanic population [9]. Cancer is one of the prominent reasons for death in Hispanics, accounting for 20% of deaths. It is expected that in the US, about 39/100 women and 40/100 men will likely develop cancer during their lifetime. Data from the American Cancer Society are presented in Table 2, showing estimated new cancer cases and estimated deaths from various types of cancer in 2022. These expectations for cancer development are based on the general population. They may vary and depend upon factors like family history, genetic vulnerability, and the kind of exposure to tobacco or radiation [18].

## 4. Current Cancer Status in the Hispanic Population in the United States

**Figure 2:**
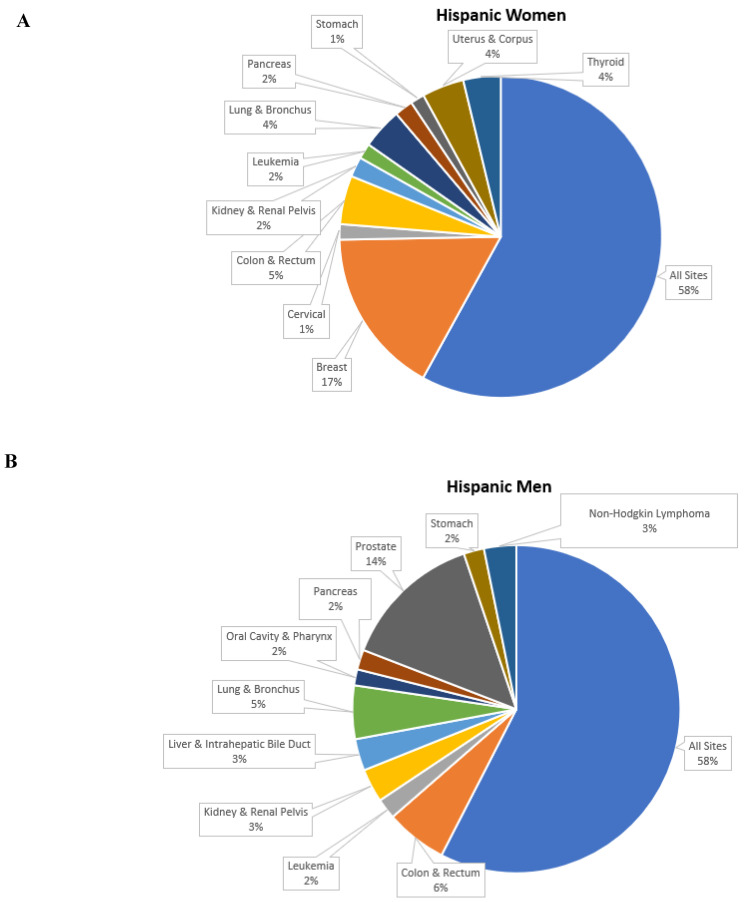

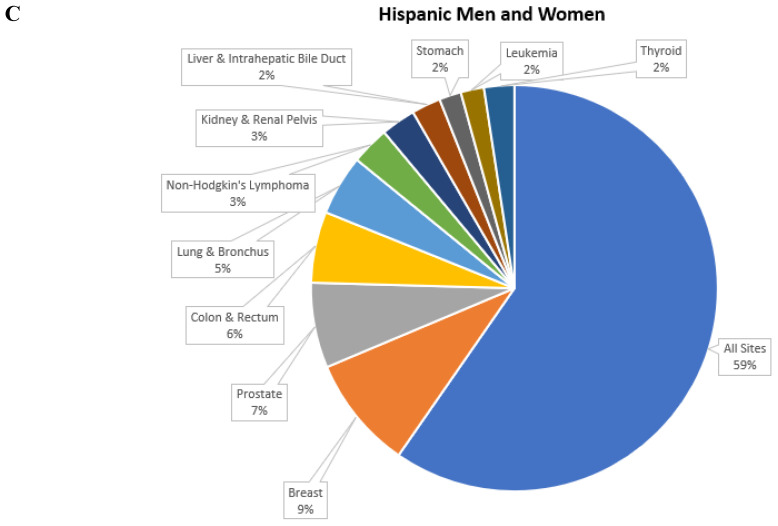
Top 10 cancer sites for the Hispanic group (2014–2018). (**A**) Cancer incidence rates per 100,000 in women. (**B**) Cancer incidence rates per 100,000 in men. (**C**) Cancer incidence rates per 100,000 in men and women.

As per the census data of 2020, the total population of Hispanics in the United States is 62.1 million. The Hispanic group represents 18.9 percent of the US population, and after the non-Hispanic whites, it is the second largest racial and ethnic group in the nation. The cancer incidence data presented in Figure 2 on the Hispanic group were collected and published by the National Cancer Institute’s and Surveillance, Epidemiology, and End Results (SEER) program.

## 5. Possible Causes of Cancer

The exact reasons for cancer development are not entirely understood; however, many factors are known to increase the risk, including possibly modifiable factors, such as obesity and tobacco use, and nonmodifiable factors, such as inherited genetic mutations. These potential risk factors may initiate or promote cancer growth simultaneously or in a sequence by complementing each other. It has been assessed that an estimated 20% of all cancers are caused by being overweight [19]. More than 100 categories of cancer and their subtypes can be observed within specific organs [5]. The stimulation of oncogenes can activate the DNA-damage response, which controls pre-malignant lesions by activating several molecules, such as DNA damage sensors, checkpoint kinase, and tumor suppressor proteins [6].

- DNA-damage sensors: These are the central regulators of the network and are activated by DNA damage and DNA replication stress. However, their DNA-damage specificities are distinct, and their functions are not redundant. These include ATM (ataxia-telangiectasia mutated), and ATR (ATM and Rad3 related) [20].
- Checkpoint kinases: Two checkpoint kinases are involved in tumors, CHK1 (Checkpoint kinase homolog 1) and CHK2. The primary function of CHK1 and CHK2 is to transmit the checkpoint signals from the proximal checkpoint kinases of the phosphatidylinositol 3-kinase group, particularly ATM and ATR [21,22].
- The tumor-suppressor protein p53: p53 is a phosphoprotein and is hardly detectable in the nucleus of normal cells [23]. With cellular stress, especially DNA damage, p53 can arrest cell cycle progression, consequently allowing the DNA to be repaired or initiate apoptosis [23,24].

## 6. Who Is at Risk?

**Figure 3:**
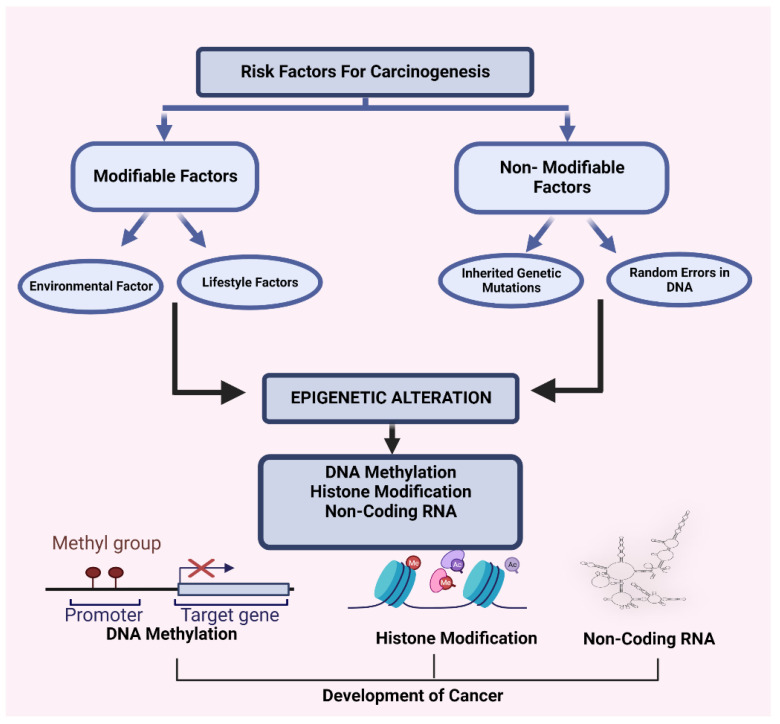
Possible hypothesis regarding the mechanism of carcinogenesis.

Extensive alterations in cancer rates are observed in different countries, and noticeable changes in cancer rates among migrating populations and rapid changes over time within countries signify that some aspects of lifestyle and environmental factors are mainly responsible for common cancers [25]. A summary of cancer risk factors is represented in Figure 3. Due to the immense effect of modifiable factors on risk, especially for the most rampant cancers, it has been estimated that 50% of cancer is preventable [26]. Many risk factors can increase the risk of developing cancer in anyone. Certain modifiable factors, including obesity [27], physical inactivity [28], alcohol consumption [29,30], an unhealthy lifestyle and diet [31], and tobacco consumption [28], are associated with an increased risk of cancer. Further, specific chemical and radiation exposure and nonmodifiable factors, such as a family history of cancer and older age, are also linked to cancer. 

## 7. Epigenetics of Cancer

Recent research has revealed that epigenetics are associated with cancer growth. Epigenetics means DNA sequence-independent covalent alterations to the nucleic acids and histones that can be inherited through cell replication and are necessary for cell fate determination [32]. The epigenetic process that modifies chromatin construction can be categorized into four central divisions: (a) DNA methylation, (b) covalent histone modifications, (c) non-covalent mechanisms, such as the integration of histone variants, and (d) nucleosome remodeling and non-coding RNAs, including microRNAs (miRNAs) [33]. Alterations to the DNA sequence result in the inactivation of tumor suppressor genes and are a significant contributor to human cancer [34]. 

DNA Methylation: DNA methylation is the most significantly studied epigenetic modification in mammals [33]. The DNA methylation model of the genome of cancer cells varies from that of normal cells [7]. DNA methylation plays a crucial role in maintaining genome stability, genomic imprinting, the inactivation of X-chromosomes in females, control of transcription, and an organism’s developmental manner [35]. DNA methylation can result in gene silencing by either inhibiting or stimulating the recruitment of regulatory proteins to DNA [33]. However, abnormal methylation of the regulatory gene promoters can cause silencing and consequent cancer development.

Histone Modification: The inherent positive charge of the essential histone proteins results in efficient binding with negatively charged DNA [36]. The N-terminal tails of these proteins are incredibly elastic. They are abundant in lysine and arginine residues, which can be highly modified by many cellular processes [37]. The N-terminal tail of histones can go through a range of posttranslational covalent modifications, such as methylation, sumoylation, acetylation, and phosphorylation ubiquitylation, on specific sites. The above-mentioned post-translational modifications are reversible and are governed by several enzymes [38,39]. These alterations regulate crucial cellular mechanisms, including transcription, replication, and repair [40]. Histone modifications can result in either activation or repression, and carcinogenesis depends upon which residues are altered and the type of modifications present [33,38,41]. 

Non-Covalent Mechanism: Non-covalent mechanisms, such as chromatin remodeling and the integration of specialized histone variants, give the cell extra tools for introducing variation into the chromatin template [42]. ATP-dependent chromatin remodeling complexes are believed to alter chromatin accessibility by modifying histone–DNA interactions, perhaps by dropping or discharging nucleosomes [42].

Non-Coding RNAs: Non-coding RNAs play a role in the epigenetic events of posttranscriptional gene modification [43,44]. MiRNA regulation is determined by several activities and depends mainly on posttranscriptional events, genomic locations, and transcription [44]. Since miRNAs regulate genes implicated in transcriptional regulation, cell proliferation, and apoptosis (the most frequent processes deregulated in cancer), modification of their expression can stimulate tumorigenesis [7]. MiRNAs can serve as either tumor suppressors or oncogenes varying upon their target genes [7]. The impact of miRNA on the epigenetic mechanism and the mutual epigenetic regulation of miRNA expression suggests that its deregulation during carcinogenesis has necessary implications for the global regulation of epigenetics and cancer [45]. 

## 8. Cancer and Dementia

Cancer and dementia are both potentially lethal and affect survival [46]. With the elderly population, cancer and dementia have become significant public health problems, causing a burden to patients, family members, the health care system, and informal caregivers [47]. In dementia, individuals cannot perform daily activities due to memory loss and multiple cognitive deficits [48]. The correlation between cancer and dementia is complicated. The well-observed direct and inverse relationship between cancer and neurodegeneration has been reported, such as with Parkinson’s disease [49,50,51]. Early cancer diagnosis and treatment improvements ensure the prolonged survival of cancer patients, accelerating the incidence of long-term side effects, such as dementia [52]. The inverse relationship between cancer and dementia shows opposite pathological mechanisms, specifically neuronal cell death due to pathological and morphological changes [53], in dementia but uncontrolled cell growth in cancer [54,55,56]. Many studies have suggested that dementia and site-specific cancers have one or more common molecular mechanisms, such as the PIN1 enzyme and signaling pathway [56,57,58]. The PIN1 gene, which is necessary for tau phosphorylation, is also overexpressed in certain cancers, and the PARK2 gene, which is responsible for producing an E3 ubiquitin ligase, may also be a tumor suppressor [59].

In addition, many factors demonstrate the link between cancer and dementia, such as age-related changes, shared risk factors, and cancer treatment effects on the brain [60]. Cognitive decline in newly diagnosed cancer patients also shares common pathologies, such as genetic susceptibility, inflammation, and oxidative stress [61,62].

## 9. Status of Cancer Disparities in the Hispanic Population

**Figure 4:**
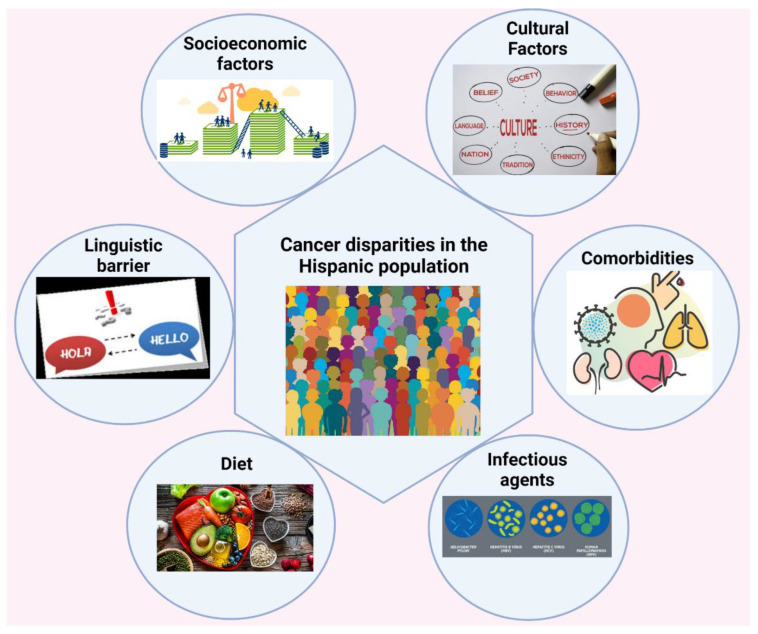
The possible cancer risk factors in the Hispanic ethnic group.

The first report on cancer disparities by race and socioeconomic status in the United States was published by “The American Cancer Society (ACS)” in 1886 [63,64]. In terms of age distribution, social standing, and immigration history, Hispanics in the United States stand apart from their non-Hispanic counterparts. Cancer risk factors, such as a sedentary lifestyle, poor diet, smoking, and obesity, are influenced by socioeconomic circumstances, which also have an impact on access to appropriate palliative care, early detection of disease, and treatment, as shown in Figure 4. Even though US Hispanic populations typically are classified as one ethnic group, they are similar to multiple cultures, but not culturally homogeneous, ethnic subgroups [65]. Hispanic populations, from the genetic perception in the United States, are considered an intermixture due to the history of their original residence, colonization of the diverse region by different European countries at other times, and different immigration histories [12]. Hispanics have higher incidence rates of cervical, gall bladder, and gastric cancer [12].

Socioeconomic factors: Socioeconomic status is a well-accepted social determinant of health [8]. The socioeconomic status affects cancer risk factors, such as physical inactivity, tobacco usage, poor nutrition, and obesity [66]. Hispanic/Latinos are more likely to live in low-income neighborhoods, which has been linked to a later stage of cancer diagnosis and worse cancer survival. Economic barriers include a lack of health insurance, low incomes, and elevated poverty rates [67]. According to the estimates, the median income for a Hispanic family in 2021 was $54,857, while for a white family, it was $81,384, which is a significant difference [8]. The Hispanic population’s access to quality healthcare services is difficult due to their lower income. There is substantial discrepancy among Hispanic subgroups; for example, Mexican Americans are the youngest, minimally educated, and the least likely to have health insurance, while Puerto Ricans are the least likely to be uninsured but have the highest proportion of unwaged people and are the most likely to live below the poverty level [68]. Socioeconomic status is the main reason for a lower breast cancer survival rate among Hispanic women compared to that in non-Hispanic whites [69].

Cultural Factors: Hispanic culture has cultural ethics that impact health behaviors and cultural aspects of care. Caregiving is largely impacted by cultural values of individuals, countries of origin, acculturation, and gender roles [67]. Gloria Juarez’s study on Perceptions of Quality of Life in Hispanic Patients with Cancer demonstrates the influence of culture. Their study showed the impact of physical, psychological, social, and spiritual well-being on the quality of life in Hispanic patients. Family and faith in God also play a significant role in Hispanic patients with cancer pain [70].

Linguistic barrier: Many studies in the past have revealed that racial and ethnic minorities receive lower-quality healthcare services, are less likely to undergo routine medical procedures, and have higher rates of morbidity and mortality compared to non-minorities despite ongoing improvements in the general health of the US population [71,72]. Language barriers, particularly in the Hispanic community, lead to the mistrust of US healthcare providers and may prevent needed medical appointments [73]. Both patients and caregivers are affected by the barrier of language [8]. Despite the Affordable Care Act’s sanction, Hispanics continue to under-enroll in many state and federal programs due to linguistic difficulties [24,74,75]. In the US, Hispanics (about 51.3%) seek a translator’s help at the time of a health care checkup [76]. 

Comorbidities: In the Hispanic population, the prevalence of type 2 diabetes is two to five-fold higher than that in non-Hispanic whites [77,78]. The related risk factors, such as hypertension, obesity, and genetic predisposition, are more likely responsible for type 2 diabetes in the Hispanic population [79]. In addition, obesity enhances the risk for numerous forms of malignancies [80]. Obesity and overweight are directly related to an increased risk of multiple cancers, such as colon and rectum [81] and breast cancer in postmenopausal women [82], while visceral obesity is strongly associated with colorectal cancer and probably associated with a greater risk of pancreatic, endometrial, and postmenopausal breast cancer [83].

Hispanic women are diagnosed with giant tumors at later stages [84], and 20% of Hispanic women die from breast cancer compared to the rate in non-Hispanic whites [85]. The high prevalence of comorbidities, such as diabetes [86] and socioeconomic status [69], are the main reasons for lower breast cancer survival rates among Hispanic women compared to those in non-Hispanic whites. Due to the immense effect of these morbidities on cancer development, we can reduce the risk of cancer due to these morbidities by changing lifestyles. The long-term impact of diabetes and obesity has been linked to chronic liver disease, mainly non-alcoholic fatty liver disease and liver cancer [87]. According to the American cancer society Liver/intrahepatic bile duct, stomach and cervical cancers were found to be higher among Hispanics than among whites.

Diet: Many factors, such as lower income and culture, are the main obstacles to healthy food choices. Studies have shown that acculturation in Hispanic American immigrants impacts an increase in dietary habits that encourage obesity, such as a higher intake of sugar and sugar beverages, higher sugar consumption, higher solid fat consumption, increased fast food intake, and more eating out [88]. Hispanics usually have saturated fat- and carbohydrate-rich food [89]. Numerous mutagenic and carcinogenic constituents are present in our food [90,91]. According to prior research, a poor diet may be a factor in 50% of all cases of breast cancer and gallbladder cancer and 70% of cases of colon cancer [92]. Low-income communities more regularly buy less costly food and higher-calorie foods, which increase the risk of T2DM [69] and obesity and contribute to cancer.

Infectious agents: About 23% of cancer cases in less developed countries and 7.4% of such instances in more developed areas are caused by infectious organisms, comprising nearly 95% of total infections, including *Helicobacter pylori*, hepatitis B virus, hepatitis C virus, and Human Papillomavirus [93]. The cancer burdens linked to these infectious agents in the United States are much higher in Hispanics than in non-Hispanic whites [94]. According to NHANES (1999–2000) published data, the seroprevalence of *H. pylori* infection in US adults is 64% in Mexicans and 21% in non-Hispanic whites, which is significantly higher in the Hispanic population [95]. First-generation Mexican migrants have a greater occurrence of HPV infection compared to that in US-born Mexican women [96]. Persistent infection with HPV is the root of nearly all anal and cervical cancers, around 40% of other genital cancers, including vaginal, penile, and vulvar cancers, and a growing percentage of neck and head cancers [97,98].

## 10. Cancer and Caregivers 

The caregiver refers to anyone who delivers care to someone who needs extra help or care. The caregiver is a broad term; anyone could be a caregiver, a family person, a friend, a respite caregiver, and a primary caregiver. Formal caregivers are professionals that have been paid to assist in completing the daily needs of individuals. In contrast, informal caregivers are often family, friends, and volunteers who are not paid for their services [8]. In addition to managing survivors and their symptoms, providing hands-on care, providing personal care and transportation, managing financial assistance, and providing emotional support, the role of caregivers evolves over time. At certain points during the illness journey, caregivers are required to take on additional responsibilities [99]. Care can be categorized as “direct care” or “indirect care,” depending on its focus [100]. This classification impacts the needs of caregiving [100]. Indirect care is provided on behalf of the patient and includes tasks like obtaining medications, transportation, scheduling and managing appointments, and assistance with the medical bills and finances. Direct care is carefully provided directly with the patient and includes activities, such as symptom management, medication administration, emotional support, and assistance with mobility or bathing [100].

Compared to people with other chronic conditions, cancer patients experience more inconsistent symptoms and toxicities from various multimodal therapies [101]. Cancer leads to fluctuations in the family’s identity, roles, and daily operations, and the impact of such changes may be intense and long-lasting, regardless of the outcome of the disease [102]. Three main areas of caregiver concern are assisting patients with the disease’s emotional consequences, the anxiety of cancer, and its outspread and managing the disruptions caused by cancer [102]. Typically, cancer care is defined by outpatient procedures, short hospitalizations, and an extended survival. Family caregivers assume accountability for the patient’s emotional and physical care, daily living activities, medication management, transportation, and communication with insurance companies and healthcare providers [100,103]. Several cancer caregivers inform cancer-related stress that can affect physical health and immune performance [104]. With the advancements in cancer diagnosis, treatment, and alleviating care, the prolonged survival of cancer patients and the duration of the caregiving phase have increased from days or weeks to months or years [103]. 

**Figure 5:**
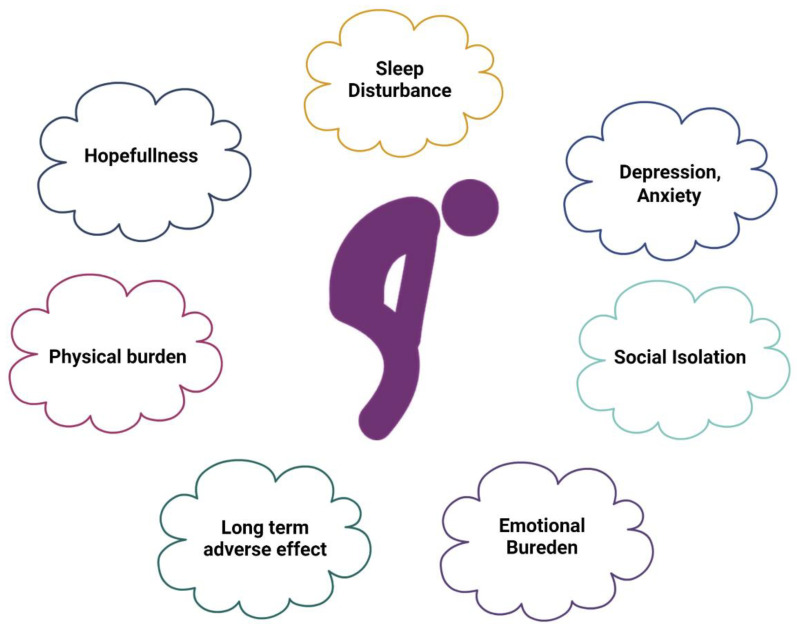
Caregivers’ burden. The caregiving burden progressively increases as the disease advances.

The health care system’s most distinctive feature is its increased emphasis on decision-making, the need for novel targeted medicines, and more challenging treatment regimens. It also moves more care away from inpatient facilities to outpatient, community-based facilities or homes [101]. As the aging population increases, the demand for caregivers and the caregiver burden will progressively increase; Figure 5 shows the caregiving burden of cancer caregivers.

## 11. Caregiver Burden in Hispanic Caregivers of Individuals with Cancer

An estimated 126,000 new cancer patients are diagnosed in the Hispanic population annually [24]. As the increasing number of Hispanics diagnosed with cancer increases, the number of caregivers will increase. While not all those who care for Hispanic cancer survivors are Hispanic themselves, the majority of them are, and they share similar cultural beliefs about health and illness [67]. Several cancer patients and survivors need care for months or years, even after active treatment, because of the late and prolonged effects of cancer and therapy [105]. The growth in the prevalence of cancer and the shift towards outpatient facility care have increased the burden on informal caregivers [106]. Because of multiple caring tasks, the spare time of informal caregivers is reduced, and their health and mental status is compromised by a lack of rest [107]. There are many cultural and contextual factors that may impact the caregiving experiences of Hispanic caregivers, and these factors may differ from those of other ethnic groups. Hispanics use fewer preventative services, more emergency or urgent care services, and other quality care indicators because they are half as likely as non-Hispanic whites to have a regular source of care or provider [67]. Employment is one important element that affects a person’s capacity to get medical treatment. A greater proportion of Hispanics than that of non-Hispanic whites engage in low-wage industries, such as agriculture, domestic work, food service, and construction [108]. Among Hispanics, providing care is frequently a defined gender role. Most Hispanic caregivers are female since caring for others is seen as a feminine function [109]. Additionally, Hispanic female caregivers are typically younger and in their 40s, less educated, and earn less than non-Hispanic whites. These factors add to the burden of caregiving, as well as psychological distress and other detrimental effects on health [110]. Numerous Hispanic caregivers report having high financial burdens and low earnings [67]. Language is another contextual factor that affects the life quality of Hispanics and their access to available resources. Spanish-speaking Hispanic people are less likely to have health insurance than non-Hispanic white people who prefer English. Access to care is also correlated with having health insurance [67].

With Hispanic culturally based values, Hispanic caregivers contribute another context of the caring component to the caregiving process [109]. There is a considerable amount of literature that shows how racial, ethnic, and cultural factors affect caring families and their experiences. A recent study determined the influence of Latino cultural values on family caregivers and found that the caregiving role and the caregiving roles of family members were influenced by Latino cultural beliefs and suggested that future studies should consider Latino cultural values as they impact various familial and health dynamics [111]. 

**Figure 6:**
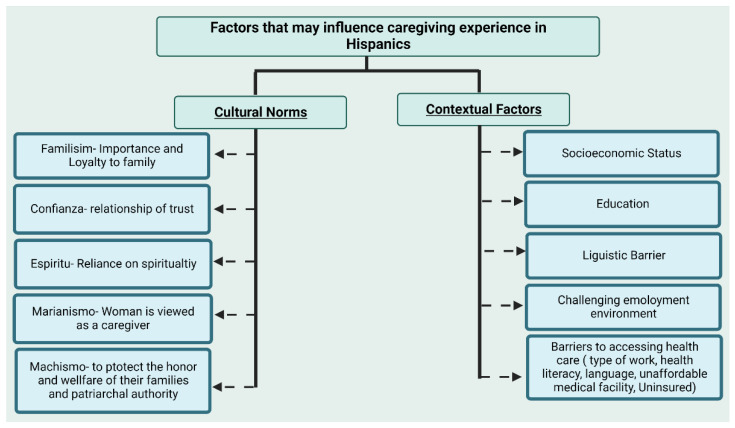
Cultural and contextual factors that may influence caregiving practices in the Hispanic population.

In Hispanic culture, the concept of familism requires them to follow their cultural norms, and the family traditionally provides care to their ill family members either in the immediate or extended family. Many cultural and contextual factors may affect the caregiving experience of Hispanic caregivers, as shown in Figure 6. Hispanic caregivers of cancer patients suffer from psychological distress and a reduced quality of life [110,112]. Because of their own emotional and physical anguish, the caregivers may not be able to provide the degree of vital support required, which has a negative impact on the cancer survivor’s quality of life [110,112]. Approximately 32% to 50% of caregivers have substantial psychological pain, suffer enhanced psychiatric morbidity, and have poorer quality of physical health [101,113]. In addition, most informal caregivers lack knowledge and competencies about cancer care, resulting in depression and low quality of life [114]. Due to traditional values, informal caregivers tend to conceal and inhibit negative emotions, avoiding entertainment and relaxation, even during their extra time [115]. With this exhausting routine, it is hard for caregivers to deal with their emotional and physical state, and this can lead to other health complications. Caregivers caring for cancer survivors have a high prevalence of anxiety (47%) and depression (42%) associated with many of the challenges [116].

Geng and colleagues’ meta-analysis of the prevalence and contributing factors of depression in caregivers of cancer patients estimated that out of 30 studies, the prevalence of depression was 42.30% and that of anxiety was 46.55 [116]. They also noticed that numerous factors might influence depression and the quality of life, including the caregiver being unemployed, the patient’s condition, duration of caregiving, spouse caregiver, financial problems, a caregiver with chronic disease, and female sex being the factors possibly associated with depression. In addition, a caregiver’s overall quality of life, the caregiver’s education level, pre-loss grief, age, and caregiver bondage with patients are negatively linked to depression in caregivers [116]. However, it is hard to describe all factors that affect the caregiver’s quality of life. Studies have noticed that being an adult or woman, having a lower income, being an ethnic minority, and a being spousal cancer caregiver may contribute to an expanded risk of caregiver burden, depression, anxiety, and sleep disturbances [117].

Felina and colleagues’ study on depression among underserved rural and urban caregivers of Latinas with breast cancer found that both the residence and acculturation were seriously linked to caregiver depression, with urban residence caregivers and less acculturated caregivers reporting higher depression. Mother caregivers had greater levels of depression compared to spouses or other caregivers. Similarly, the urban residence was consistently linked to more significant depression regardless of additional covariates [118]. A recent study analyzed data from the Cancer Care Outcomes Research and Surveillance Consortium and found that compared to that in non-Hispanic white caregivers, Hispanic and black cancer caregivers provide more care and report a greater financial burden, but they also face lower or comparable social/emotional and health difficulties. Black–white burden inequalities are partially explained by racial differences in caregivers’ social support systems and levels of caregiving readiness. The study suggested that the growing financial burden on black and Hispanic caregivers should be the subject of research and legislation [119], as shown in Figure 5.

## 12. End-of-Life Caregiving

Caregiving at the end of life demands much more than the functional tasks of assisting a person with cancer. End-of-life care services offer emotional and physical support for people with terminal illnesses during their final stages of life. Based on hospice use rates reported by The National Hospice and Palliative Care Organization, the hospice admission rate for Latino/Hispanic deaths is 38%, while the admission rate among non-Hispanic whites is 82% [120]. End-of-life care among Hispanics is mainly at home rather than in-facility, which is evidence of cultural preference for home-based end-of-life care [121]. These data proved that Hispanics prefer informal care. Many Latino individuals find themselves under immense emotional and financial strain when challenged with the end of the life of a family member, combined with the associated caregiving demands [122]. In Latinos, death and dying concerns may be considered taboo, potentially restricting discussions about the burdens linked to increased levels of care because of functional deterioration at the end of life [123].

## 13. Caregiving with Advanced Cancer

The lives of patients and their families become even more unsettled when the patient has a progressive illness, terrible symptoms, or an uncertain survival possibility [124,125]. Huiwen and colleagues’ study also found rural–urban disparities in caregivers of older adults with advanced cancer. Caregivers are at risk of caregiving burden, specifically those from rural areas and with a lower education [126]. Serge Dumont and colleagues’ study found that family caregivers of patients in the advanced stages of cancer live through a high level of psychological distress, which grows significantly as the patient loses autonomy [123]. In addition, a high distress index was considerably linked to the caregiver’s burden, the patient’s young age, the patient’s indications, the caregiver’s young age and gender, a poor perception of their health, and disappointment with emotional and tangible support [123]. 

## 14. Adult Cancer Survivors and Their Caregivers

The degree to which family caregivers experience both positive and negative aspects of caregiving may impair their capacity to provide for the survivor. Their multifaceted quality of life (QOL) is related to that ability [127]. Caregivers of cancer survivors describe a variety of issues as a result of their caring experiences, such as conflicting social responsibilities, restrictions on their activities, tension in their marriages and families, psychological anguish, and deteriorated physical health [100,128,129]. Complications in maintaining or forming new social relationships are regularly mentioned as one of the most significant long-term issues for adolescent and young adult (AYA) cancer survivors [130]. Cancer survivorship rates are increasing, and cancer patients are living longer and complete lives; as a consequence of these encouraging medical outcomes, caregivers can face extra responsibilities and an increased burden in caring for a loved one with cancer [131]. According to Terry and colleagues’ research, Hispanic cancer survivors’ caregivers are impacted by contextual and cultural factors. Hispanic caregivers experience poor health through caregiving and have unmet needs for knowledge and emotional support [67].

## 15. Interventions to Reduce the Caregiving Burden in Hispanic Caregivers

The cancer diagnosis not only affects the diagnosed patient but also profoundly affects the family of the patient. The patient and the family caregivers form a social unit that is adversely affected throughout the journey of this disease: starting from diagnoses and ending at the end of life [99,132,133]. Family caregivers not only suffer from the cancer diagnoses of their loved ones but are also neglected by most healthcare systems and providers who are merely focusing on the needs of the patients and giving minimal attention to the caregiver’s needs [134].

Culturally sensitive interventions: Hispanic caregivers bring a different viewpoint to the caring process by incorporating culturally based Hispanic values [109,135]. Many studies show the impact of cultural beliefs and perspectives on cancer patients and their caregivers [70,136,137,138], and culturally sensitive interventions can be a powerful resource. First-generation Hispanics (meaning they were foreign-born) differ from second-generation Hispanics in diet, language use, acculturation, and other features, which result in differential health outcomes [138]. Hispanic caregivers bring a different viewpoint to the caring process by incorporating culturally based Hispanic values [139]. Culturally sensitive interventions are required to support Hispanic patients with cancer and their family caregiver in managing their care, directing the healthcare system, and reducing disparities in healthcare outcomes for Hispanics with cancer. 

Self-care for caregivers: The caregivers may experience specific symptoms of stress, depression, and anxiety while taking care of the patient, which can affect their personal life drastically, such as feeling exhausted, frequent illness, feeling irritated, sleeplessness, loss of appetite, etc., and some of the following steps can be used to cope with such symptoms: make a routine for exercise and relaxing practices, such as yoga and meditation, limit/stop the consumption of alcohol and tobacco, organize some activities with family and friends to make yourself comfortable and happy, take help from related support groups or communities, have a healthy diet, take care of your body and be kind to yourself, get help from relatives, friends, or any religious and community groups, divide responsibilities among family members, watch movies, and listen to feel-good music.

Use of Technology: A recent study discussed disparities among cancer patients using telemedicine services during the COVID-19 pandemic and found that as compared to non-Hispanic white, Hispanic patients were 14% less likely to use telehealth services. In the same way, Spanish-speaking cancer patients were found to be 29% less likely to use telehealth services as compared to their English-speaking counterparts [140]. Innovations in information technology and medical informatics have created new opportunities to provide new healthcare models [141], but the reluctance of Hispanic patients/caregivers to use telehealth services keep them devoid of available resources. Establishing more robust, interactive platforms that evaluate data from multiple sources will decrease the complications of health and social service coordination and care management [142]. Chi and colleagues’ systematic review of telehealth and caregivers recognized six technology categories: education, consultation, psychosocial/cognitive behavioral therapy (including problem-solving training), data collection and observing clinical care delivery, and social support [143]. In cancer treatment, an encouraging effort is being made to provide technology-based care to enhance patient outcomes. The Center for Health Enhancement Systems Studies is an organization of the University of Wisconsin–Madison that implements research and develops innovative health systems to optimize a person’s health behaviors, quality of life, and access to healthcare services [144]. Culturally and linguistically appropriate technology can support and enhance the health literacy of Hispanic caregivers and accelerate better coping.

Use of recommended Screening Tests for Cancer: Even with innovations in screening and treatment during the past several decades, cervical and breast cancer remains a significant health problem for Hispanic women, as many women have never had a Pap smear or mammogram or are not tested repeatedly. Hispanics have lower cervical, breast, and colon cancer screening rates than non-Hispanics [145]. They generally have low participation rates in cancer screening and other prevention programs. Hispanic populations typically have low participation rates in cancer screening and other prevention programs [12]. Despite the consequences of acculturation, Hispanics are more likely to obtain suggested cancer screenings if they have health insurance and an authoritative source of health care [146,147,148]. Several extrinsic determinants, such as a lack of insurance, the usual source of health care, acculturation, and socioeconomic status, while intrinsic determinants, such as salient beliefs about cervical cancer and perception of vulnerability to cervical cancer, affect the screening practice of Hispanic women [149]. Regular screening, such as for cervical cancer Pap smear testing, breast cancer mammograms, and breast magnetic resonance imaging, are the best methods for early cancer detection and the initiation of treatment.

Healthy lifestyle: In the United States, an estimated 14% to 20% of all cancers are caused by overweight and obesity [150]. A meta-analysis, including three studies, has demonstrated that a post-diagnostic low-fat diet can reduced the risk of breast cancer reappearance and improve breast cancer survival [151]. Vegetable and fruit consumption reduces the risk of obesity [152]. A systemic review showed that calorie restriction resulted in a 75.5% decline in tumor incidence with various tumor models [153]. Exercise can be applied as a target of cancer-suppressor gene therapy to change cancer metabolism and inhibit Warburg anaerobic glycolysis [154]. Studies have shown that exercise decreases breast cancer risk from 15% to 20% and reduces colorectal cancer by 24% [155]. Studies have shown that physical exercise and a healthy diet can reduce cancer incidence. It can prevent cancer growth and metastasis and improve patient quality of life.

The Affordable Care Act for Hispanics/Latinos: In the United States, racial/ethnic disparities in health care are pervasive and well-documented [156]. Health inequities—systematic, avoidable, and unfair variations in the health of groups and communities holding unequal social positions—can lead to health disparities [111]. Despite the numerous efforts that have been made to improve health in the United States, these racial and ethnic disparities are likely the most persistent health inequities over time [157,158] Hispanics/Latinos have a higher prevalence of unmet supportive care needs and limited access to information [159,160]. Implementing the Affordable Care Act may improve some of these health-related disparities. Many policies, such as a flexible employee leave policy, emergency care and paid or subsidized respite services, paid family and medical leaves, flexible scheduling of work hours, anti-discrimination protection/laws, health spending accounts, critical Illness and disability insurance, and Retirement Accounts for Hispanics can help and safeguard caregivers at the local, state, and federal levels. 

## 16. Conclusions

Hispanics are the fastest growing minority in the US, and cancer is one of the leading causes of deaths in this ethnic group. The genetically admixed Hispanic population of the US presents opportunities for elucidating the effects of genetics, environment, and lifestyle on cancer risk and identifying novel risk factors due to secular trends in environmental exposures and lifestyle/behavioral practices connected to immigration and acculturation. Hispanic cancer sufferers anticipate receiving care from their own families during the period of the illness and even after surviving cancer. Although caregiving may be the same in all ethnic groups, how family members define cultural values and beliefs influence caregiving. Cultural values and beliefs substantially affect how Mexican caregivers perceive their appraisal of the caregiver role and the caregiving experience. Hispanic caregivers have unmet informational and emotional support/needs and experience worse health when providing care. Circumstances and cultural beliefs may affect the caring experience for Hispanic caregivers. These should be evaluated by healthcare professionals as part of the planning for high-quality care and management. The Hispanic/Latino population in the US continues to face many challenges, such as higher uninsured rates, poor access to health care, a lack of representation in oncology clinical trials, discrimination, and many other factors. The growing financial burden on Hispanic caregivers should be the subject of research and legislation. 

Future investigation is needed to understand the reasons for the increased burden on caregivers in ethnic groups of the US. The outcomes of well-thought-out studies may help to develop appropriate strategies and remedies for boosting the beneficial effects of caregiving. Many interventions based on technology and the internet can empower Hispanic caregivers by providing them with a platform of useful and accessible resources. Many previous studies on the interventions for caregivers were mainly focused on the use of the internet, management of pain and stress in caregivers, their basic needs, and caregiver’s education. Although the studies have shown that technological interventions are tools that can greatly help and alleviate caregivers’ burden, a still limited amount of research has been performed on technological-based interventions for informal caregivers. Mobile applications, wearable gadgets with an understandable interface, and software/websites in Spanish can provide much-needed support to the Hispanic community, as the language barrier is the major hurdle in access to healthcare facilities.

## Figures and Tables

**Table 1:** List of Global Cancer Data of Top five Countries.

Cancer Mortality in Women	Global Cancer Incidence: Both Sexes	Cancer Incidence in Men	Cancer Incidence in Women	Global Cancer Mortality: Both Sexes	Cancer Mortality in Men
Zimbabwe	Denmark	Hungary	Denmark	Mongolia	Mongolia
Mongolia	Ireland	Latvia	Belgium	Serbia	Hungary
Samoa	Belgium	France	Ireland	Hungary	Slovakia
Malawi	Hungary	Lithuania	Netherlands	Montenegro	Serbia
Serbia	France	Slovakia	-	Slovakia	Montenegro

**Table 2:** Estimated new cancer cases reported and deaths occurring due to various types of cancer in the US in the current year.

Estimated Number of New Cases and New Deaths for Both Sexes—2022		
Type of Cancer	Estimated New Cases (Both Sexes)	Estimated Deaths (Both Sexes)
All Sites	1,918,030	609,360
Respiratory system	254,850	135,360
Digestive system	343,040	171,920
Breast	290,560	43,780
Leukemia	60,650	24,000
Myeloma	34,470	12,640
Genital system	395,600	68,260
Oral cavity & pharynx	54,000	11,230
Lymphoma	89,010	21,170
Urinary system	164,190	31,990
Brain and other nervous systems	25,050	18,280

## Data Availability

Not applicable.

## Abbreviations

AARP	American Association of Retired Persons
ACS	American Cancer Society
AYA	Adolescent and young adult
CDC	Centers for Disease Control
CHK1	Checkpoint kinase homolog 1
CHK2	Checkpoint kinase homolog 2
HMT	Histone methyltransferases
miRNAs	MicroRNAs
NHW	Non-Hispanic whites
PTM	Post-transcriptional modifications
QOL	Quality of life
ROS	Reactive oxygen species
SNP	Single nucleotide polymorphism
T2DM	Type 2 diabetes mellitus
US	United States
WHO	World Health Organization
